# RISK FACTORS, PREVENTION, AND TREATMENT OF INFECTIONS RELATED TO TOTAL HIP ARTHROPLASTY: SYNTHESIS OF CLINICAL EVIDENCE

**DOI:** 10.1590/1413-785220253306e290069

**Published:** 2025-11-10

**Authors:** Tiago Afonso Silva Abati, Marco Antonio Bononi, Rafael Costa Lima, Israel Scholtz Veiga

**Affiliations:** 1 Hospital de Base Ary Pinheiro (HBAP), Servico de Ortopedia e Traumatologia, Porto Velho, Rondonia, RO, Brazil.; 2 Hospital Nossa Senhora do Rocio, Servico de Ortopedia e Traumatologia, Campo largo, Parana, PR, Brazil.

**Keywords:** Arthroplasty, Replacement, Hip, Prosthesis-Related Infections, Postoperative Complications, Therapeutics, Artroplastia Total de Quadril, Infecções Relacionadas à Prótese, Complicações Pós-Operatórias, Terapêutica

## Abstract

Total hip arthroplasty (THA) is a complex surgery and is indicated for the treatment of degenerative diseases such as osteoarthritis, rheumatoid arthritis and osteonecrosis, as well as femoral neck fractures. This procedure aims to restore mobility, relieve pain and improve patients’ quality of life. However, infections, especially periprosthetic joint infection (PJI), are serious complications that can compromise the success of the surgery. To identify risk factors, as well as methods of preventing and treating infections in THA. An integrative literature review was carried out, selecting clinical trials published in the last 10 years that addressed the proposed topic, using the following search strategy in the PUBMED database: hip[title] AND arthroplasty[title] AND infec*[title]. The analysis involved reading and discussing 12 articles, which addressed different aspects of infection prevention and management in THA. Although some interventions, such as collagen sponges with gentamicin and triclosan-coated sutures, have not significantly reduced the incidence of infections, others, such as closed incisional negative pressure therapy (ciNPWT) and washing with diluted betadine, have shown promise in certain contexts. Diagnostic accuracy, especially for coagulase-negative staphylococci, still presents challenges, highlighting the need for advances in diagnostic and therapeutic methods. Thus, despite advances, the prevention and management of infections in THA still require improvement, and interventions must be carefully evaluated to ensure the effectiveness and safety of the procedure. ***Level of Evidence IV; Evidence from Descriptive (non-experimental) or Qualitative Studies***.

## RESUMO

A artroplastia total de quadril (ATQ) é uma cirurgia complexa, sendo indicada para tratamento de doenças degenerativas como osteoartrite, artrite reumatoide e osteonecrose, além de fraturas do colo do fêmur. Este procedimento visa restaurar a mobilidade, aliviar a dor e melhorar a qualidade de vida dos pacientes. No entanto, as infecções, especialmente a infecção articular periprotética (IAP), são complicações graves que podem comprometer o sucesso da cirurgia. Identificar fatores de risco, assim como métodos de prevenção e tratamento das infecções em ATQ. Foi realizada uma revisão integrativa da literatura, selecionando ensaios clínicos publicados nos últimos 10 anos que abordavam o tema proposto, utilizando a seguinte estratégia de busca na base de dados PUBMED: hip[title] AND arthroplasty[title] AND infec*[title]. A análise envolveu a leitura e discussão de 12 artigos, que abordaram diferentes aspectos da prevenção e manejo das infecções em ATQ. Embora algumas intervenções, como as esponjas de colágeno com gentamicina e suturas revestidas com triclosan, não tenham reduzido significativamente a incidência de infecções, outras, como a terapia de pressão negativa incisional fechada (ciNPWT) e a lavagem com betadine diluído, mostraram-se promissoras em determinados contextos. A precisão diagnóstica, especialmente para estafilococos coagulase-negativos, ainda apresenta desafios, destacando a necessidade de avanços nos métodos diagnósticos e terapêuticos. Sendo assim, apesar dos avanços, a prevenção e o manejo das infecções em ATQ ainda requerem aprimoramento, e as intervenções devem ser cuidadosamente avaliadas para garantir a eficácia e segurança do procedimento. ***Nível de Evidência IV; Evidências de Estudos Descritivos (não experimentais) ou com Abordagem Qualitativa***.

## INTRODUCTION

Total hip arthroplasty (THA) is a highly complex surgical procedure designed to replace the damaged hip joint with artificial prosthetic components. The procedure aims to restore mobility, relieve pain, and improve the quality of life of patients suffering from advanced joint pathologies. The surgical technique involves removing the compromised articular surfaces and replacing them with a prosthesis consisting of a femoral stem, a femoral head, and an acetabular component, which together reconstitute joint functionality.^1^

The indications for total hip arthroplasty are varied and include primarily degenerative diseases such as osteoarthritis, rheumatoid arthritis, and osteonecrosis, as well as femoral neck fractures that cannot be adequately managed by other methods. In addition, congenital deformities and sequelae of dislocations or trauma may also require hip joint replacement. THA is often considered when conservative therapeutic options no longer provide sufficient pain relief or when there is significant functional limitation.^2^

Among the complications associated with total hip arthroplasty, infection is one of the most severe and challenging. Periprosthetic infection may occur at different postoperative periods, ranging from early infections, shortly after surgery, to late infections, years after prosthesis implantation. This complication is particularly concerning because it may compromise surgical success, require additional interventions, and in severe cases, lead to prosthesis removal. Effective management of this complication requires a multidisciplinary approach, including early diagnosis, preventive strategies, and specific treatments, which may range from antibiotic therapy to complex surgical revisions.^3^

Given the severity of potential complications and the complexity of the procedure, it is imperative that risk factors be carefully evaluated and preventive measures rigorously implemented.^4^ Furthermore, the establishment of effective treatment protocols in cases of infection is essential to optimize THA outcomes.

## MATERIALS AND METHODS

This study was designed as an integrative literature review. Study selection was performed in the PUBMED database using the following search strategy: hip[title] AND arthroplasty[title] AND infec*[title]. Only clinical trials published in the last 10 years were included. The research question that guided this review was: "*What are the risk factors associated with infections in THA, as well as their diagnostic and treatment methods?*"

The review process was conducted in six sequential stages: formulation of the research question, identification of relevant studies in the literature, data collection from the specified database, critical and detailed analysis of the selected studies, discussion of the findings, and finally, preparation and presentation of the integrative review, as proposed by Souza et al.^5^

## RESULTS

The initial search retrieved 12 articles that met the search strategy defined for this review. After screening titles and abstracts, all identified articles were selected. Subsequently, the studies were read in full, summarized, and discussed, following a chronological order based on their year of publication.

## DISCUSSION

Surgical site infection (SSI) has been widely recognized as one of the most feared complications in surgery, particularly in hip arthroplasty, as highlighted by Westberg et al.^6^ Their study, conducted between February 2011 and July 2013, investigated the effectiveness of collagen sponges containing gentamicin in preventing SSI in elderly patients undergoing hemiarthroplasty after femoral neck fracture. The results did not reveal a statistically significant difference in SSI incidence between the gentamicin-collagen group and the control group, indicating that the use of such sponges did not reduce infection rates.

Similarly, González-Vélez et al.^7^ emphasized the severity of SSIs, especially in hip arthroplasty, by analyzing the excessive direct costs associated with these infections. In a case-control study conducted at Hospital Universitario Ramon y Cajal, Spain, they identified that infections related to methicillin-resistant *Staphylococcus aureus* increased costs by 134%, reinforcing the need for preventive interventions to minimize both financial and clinical impacts of such infections.

Ibrahim et al.,^8^ addressed another critical aspect of post-arthroplasty complications: periprosthetic joint infection (PJI). In a study comparing the treatment of patients with negative and positive cultures, they found that culture-negative PJIs presented particular challenges, but adherence to strict protocols allowed reinfection rates similar to those observed in culture-positive patients, underscoring the importance of adherence to well-defined therapeutic strategies.

Sprowson et al.^9^ investigated whether triclosan-coated sutures could reduce SSI incidence in patients undergoing total hip and knee arthroplasty. The study, involving 2,546 patients, found no significant evidence that these antimicrobial sutures reduced infection rates, suggesting that the introduction of such technologies must be carefully evaluated before routine implementation.

Closed incisional negative pressure wound therapy (ciNPWT) was assessed by Newman et al.^10^ in a study comparing its effectiveness with traditional dressings in patients undergoing revision arthroplasty. The results demonstrated a significant reduction in wound complications and reoperations in the ciNPWT group, indicating that this technique may be beneficial for high-risk patients.

Keeney et al.^11^ also explored the use of negative pressure wound therapy (iNPWT), but focused on patients undergoing total lower extremity arthroplasty. They observed that although iNPWT could increase initial wound drainage, the devices were effective in reducing complications in patients with elevated body mass index, particularly after total knee arthroplasty.

In the diagnostic field, Kleiss et al.^12^ evaluated the accuracy of the synovial alpha-defensin enzyme immunoassay for diagnosing PJI. Despite high specificity rates, the test failed to correctly detect some infections caused by coagulase-negative staphylococci, revealing that additional diagnostic methods are still needed to ensure accurate detection in all cases.

Calkins et al.^13^ investigated whether diluted betadine lavage could reduce postoperative PJI rates compared with saline lavage. The results indicated a significant reduction in infections in the betadine group, suggesting that this practice may serve as a simple and effective preventive measure in aseptic revisions.

Yang et al.^14^ conducted a study analyzing the impact of a three-month course of targeted oral antibiotics in patients undergoing revision for chronic prosthetic joint infections. Their findings showed a significant reduction in reinfection rates among patients who received antibiotics, highlighting the effectiveness of prolonged treatment in preventing relapse.

In the study by et al.^15^ outcomes of static versus articulating spacers were compared in patients with PJI undergoing two-stage revision arthroplasty. The authors concluded that although hospital stays were longer for patients with static spacers, there was no significant difference in operative time during the second-stage reimplantation, indicating that both methods are comparable in terms of effectiveness for PJI treatment.

Finally, Amini et al.^16^ reported that in their research on the use of antibiotic-loaded cement spacers, antibiotic concentrations in the joints were not significantly affected by the use of drainage devices. This suggests that such devices do not compromise the effectiveness of spacers in preventing infection during two-stage revision arthroplasty.

## CONCLUSION

The studies evaluated demonstrate consensus regarding the severity of infections related to hip arthroplasty; however, they diverge on the effectiveness of the preventive interventions tested. While some methods, such as gentamicin-loaded collagen sponges and triclosan-coated sutures, did not show significant impact in reducing infections, other approaches, such as closed incisional negative pressure wound therapy (ciNPWT) and diluted betadine lavage, yielded promising results in specific contexts. Diagnostic analyses, although advancing in accuracy, still reveal limitations—particularly in the detection of infections caused by coagulase-negative staphylococci—highlighting the ongoing need for improvement in diagnostic and therapeutic methods.
